# Estimating annual deaths from stroke in adults under 70 years of age in Freetown Sierra Leone: A comparative analysis of a hospital-based stroke register and a population-based verbal autopsy study

**DOI:** 10.1177/17474930251367517

**Published:** 2025-08-05

**Authors:** Daniel Youkee, Marina Soley-Bori, Gibrilla Fadlu Deen, Prabhat Jha, Anteneh Assalif, Charles Wolfe, Catherine Sackley, Zainab Conteh, Julia Fox-Rushby, Iain Marshall

**Affiliations:** 1 School of Life Course and Population Health Sciences, King’s College London, London, UK; 2 Stroke Research Group, Neurology Division, Neuroscience Institute, University of Cape Town, Cape Town, South Africa; 3 College of Medicine and Allied Health Sciences, University of Sierra Leone, Freetown, Sierra Leone; 4 Centre for Global Health Research, Unity Health Toronto and Dalla Lana School of Public Health, University of Toronto, Toronto, ON, Canada; 5 School of Health Science, University of Nottingham, Nottingham, UK

**Keywords:** Stroke, register, completeness, selection bias, epidemiology, incidence

## Abstract

**Background:**

In Sub-Saharan Africa (SSA), most stroke epidemiological data comes from hospital-based registers, which are prone to selection bias, and data may be unrepresentative of stroke burden at the population level. The degree of incompleteness and bias in hospital-based registers has been assessed in high-income countries but not in an SSA country.

**Aims:**

The study describes and compares estimates of annual deaths from stroke under 70 years of age, from a hospital-based stroke register and a population-based verbal autopsy (VA) study. We describe the sociodemographic and clinical differences between patients captured and those missed by a hospital-based register and estimate the completeness of a hospital-based register in Sierra Leone.

**Methods:**

We compared people under 70 years of age who died from stroke in the Stroke in Sierra Leone (SISLE) prospective longitudinal hospital-based register to the Healthy Sierra Leone (HEAL-SL) population-based VA study which sampled 2.5% of households in the Western Area. We included participants from SISLE and HEAL-SL who died within the same dates (1st May 2019 until 30th September 2021) and geographical area. We conducted data linkage using probabilistic matching and manual clerical review by two authors. To assess selection bias, we used univariable analysis to identify variables associated with capture by the hospital register. To estimate annual deaths from stroke, two-source capture-recapture analysis was conducted using the Lincoln-Petersen-Chapman estimator. Estimates of completeness were adjusted for undermatching and for the positive predictive value of VA for stroke diagnosis. Deaths rates from stroke were calculated as deaths per 100,000 individuals, with population estimates sourced from the 2021 Mid-term Population and Housing Census.

**Results:**

A total of 345 participants were identified in the SISLE dataset, 46 in the VA dataset, and 4 in both datasets. Excluding individuals captured in both datasets, individuals identified by VA had a mean age of 58 years compared to 55 years in SISLE (*p* = 0.07), 59.5% were male compared to 50.7% in SISLE (*p* = 0.28), and 52.3% had no formal education compared to 39.0% (*p* = 0.09) in SISLE. Individuals identified by VA were more likely to be employed 36.7% vs 59.5% (*p* = 0.002), were less likely to have sought formal healthcare 48.5% vs 100% (*p* < 0.001), more likely to have died suddenly 14.3% vs 4.1% (*p* < 0.001), and less likely to have died in hospital 19.0% vs 67.5%. Estimates of annual deaths from stroke using capture-recapture methods ranged from 41 to 106/100,000. The completeness of SISLE register for fatal stroke ranged from 10.6% (95% CI: 9.6%–11.7%) to 27.2% (95% CI: 24.8%–30.0%).

**Discussion:**

In this setting, a hospital-based stroke register underestimated deaths from stroke in adults younger than 70 years to a much greater degree than estimates from high-income country settings. For people who died from SISLE, employed people, people who did not seek formal healthcare, and people who died within 24 hours were less likely to be included in the hospital-based stroke register. Investment in routine death registration systems and population-based stroke surveillance is essential to provide accurate estimates of population-level stroke burden in our setting.

## Graphical abstract

**Graphical abstract:**
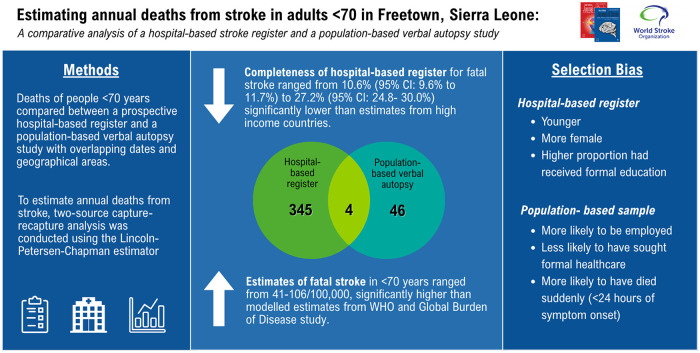

## Introduction

There is an absence of population-based stroke epidemiological research in Sub-Saharan Africa (SSA), and stroke surveillance largely relies on hospital-based stroke registers.^1^ Hospital-based registers can provide locally relevant data to estimate the impact of stroke and plan health service improvements. However, they provide a biased representation of the true population level impact of the disease,^2^ accentuated in locations with inequitable access to health care. Hospital-based registries underestimate the total incidence of stroke,^3^ missing people who do not seek care and people who die before reaching hospital.^4^ Many epidemiological studies of stroke in SSA are limited by the identification of fatal events in the community compounded by the scarcity of routine death registration.^5^ Less than one-third of deaths in Africa are registered,^6^ with only four African countries having a death registration system that meets international standards. In the absence of standardized death registration systems, VA methods^7^ are an alternative to determine cause of deaths that occur in the community. The accuracy of VA for stroke is likely higher than for other conditions, due to unilateral weakness being a relatively unique clinical sign.^8^ Sensitivity and positive predictive values (PPV) of VA for stroke, from Ethiopia, China and South Africa, have ranged from 68% to 87% and 52% to 89%, respectively, depending on the methodology and gold standard used.^9–11^

While it is established that hospital-based registers underestimate the number of people having a stroke, it is unclear who they miss in a low-income country setting. The selection bias of hospital-based stroke registers has been assessed in European countries, but not yet in a country in SSA. Analysis of hospital-based registers in Sweden showed they missed patients with mild stroke, patients in long-term care facilities, and those with sudden severe stroke who died before seeking hospital care.^12^ In France, Giroud and colleagues compared 12-month recruitment from a population-based stroke register, using hospitals, GP clinics, radiology centers, and death certifications as sources of case ascertainment, to a single hospital stroke register. The hospital-based register patients were younger, had a higher proportion of intracerebral hemorrhage and a higher case fatality compared to the population-based study.^13^ A similar study of patients with intracerebral hemorrhage, comparing a hospital-based register to the Dijon population-based study, found the two cohorts to be similar, except patients captured by the hospital-based register were younger.^14^ A population-based stroke register in South London has demonstrated differences in case ascertainment source by ethnicity,^15^ with patients of black ethnicity and lower socioeconomic status having lower odds of being admitted to hospital.^16^

In this study, we compare two data sources that captured deaths from stroke in Sierra Leone: the Stroke in Sierra Leone (SISLE) register and the Healthy Sierra Leone (HEAL-SL) study. SISLE is a prospective longitudinal hospital-based register conducted at two tertiary hospitals from 2019 to 2021.^17^ The SISLE register provides high quality stroke diagnosis and long term follow up of participants, however it is prone to selection bias as it only includes people who sought care at either of the two hospitals. The hospitals are based in the Western Area where the capital, Freetown, is located, and their patient population has higher rates of education^18^ and shorter distances from home to hospital, compared to the rest of the country. They are the only tertiary medical government hospitals, In the Western Area, yet private health care facilities and government primary and secondary healthcare facilities are also located in the Western Area, which were not included in the SISLE study. To promote recruitment onto the register, regular outreach visits were conducted to major facilities, study inclusion criteria promoted among the medical community and all patient investigations were funded by the study. During an overlapping time period, the HEAL-SL study, a nationwide VA study, was conducted in Sierra Leone.^19^ HEAL-SL provides population-based estimates through random household sampling covering 5% of the population of Sierra Leone. To assess selection bias we compare socio-demographics of participants with fatal stroke in the two datasets and identify variables associated with inclusion on the hospital-based register. This study then links these two datasets, providing estimates of annual deaths from stroke in Freetown, using capture-recapture methodology which can be used to estimate true population incidence underlying register data.^20^

## Methods

### Data sources

SISLE registry is a prospective longitudinal hospital-based register conducted at two tertiary hospitals from 1st May 2019 until 30th September 2021 at Connaught Teaching Hospital and at 34th Military Hospital from 1st February 2021 until 2nd September 2021.^17^ All patients aged 18 years and over meeting the WHO International Classification of Diseases-10 (ICD-10) definition of stroke were included. The study methods and the health care setting have been previously described.^21^ All stroke subtypes were included: ischaemic (ICD63), intracerebral hemorrhage (ICD61), subarachnoid hemorrhages (ICD60), and unspecified stroke types (ICD62).^19^ Multiple overlapping sources of notification were used inside the hospital including: accident and emergency admission logs; radiology request notes, ward admission logs; and physiotherapy referral logs. Daily ward rounds were performed by the clinical research team in the acute medical admission unit, and daily ward rounds of the four medical wards, private wards and the intensive care unit. The study funded access to neuroimaging, which was undertaken by 857 (87%) of the cohort. Diagnosis of stroke and stroke subtype were made by a consultant stroke physician. The cohort includes 986 participants with confirmed stroke, with participants followed up at 90 days, 1 year, and 2 years post stroke.

HEAL-SL is an electronic verbal autopsy (VA) study of ascertained deaths of individuals younger than 70 years in 678 randomly selected villages and urban blocks throughout the country.^19^ The random sampling method aims to be representative of the population and covers 5% of the total population.^19^ The electronic VA uses the 2016 WHO Verbal Autopsy Standard tool, and two trained physicians independently assigned causes of death according to the ICD-10.

### Creation of an overlapping dataset

The two datasets were restricted to create an overlapping dataset, including participants resident in the Western Area Urban and Rural districts, the catchment area of the two hospitals in SISLE, who died from stroke from 1st May 2019 until 30th September 2021.

As HEAL-SL only investigated deaths in those younger than 70 years, the SISLE dataset was restricted to include only fatal strokes in the same age group. From HEAL-SL, we included participants with a diagnosis of ICD I60 Subarachnoid hemorrhage; ICD61 Intracerebral Hemorrhage; ICDI62 other intracranial hemorrhage; ICD63 cerebral infarction; ICD64 stroke type not specified; and ICD I69 sequelae of cerebrovascular disease. From SISLE registry, we included participants with confirmed stroke, as diagnosed by a stroke physician after review of clinical notes and neuroimaging.

Dataset harmonization was conducted to ensure alignment between variables in the datasets.^22^ For example, forename and surname were separated, and any middle names were entered into a new middle name variable. Data was then standardized to ensure that all linkage variables followed the same format to improve character string similarity across the datasets. Prefixes (Dr, Mr, Alhaji, Mummy, Sheikh, Reverend) were removed from the names. Dates were formatted as day, month, year. Then the coding of data within each variable was standardized; for example, female was coded as 0 and male as 1.

### Probabilistic matching

We conducted probabilistic matching followed by manual clerical review, to identify individuals recorded in both datasets. Spelling mistakes for name and address variables have been reported as present in 22% of SSA routinely collected datasets.^23^ In Sierra Leone, there is also variation in how names are spelt and how ages and geographical location are described.^24^ In Sierra Leone, certain names are very common; an analysis of 66,563 records in the Sierra Leone Ebola Database reported that the most frequent surname was used by 18.2% records and the most frequent 20 surnames accounted for 74.1%.^24^ Therefore, we used an extension^25^ of the Fellegi–Sunter-based method^26^ to compute a weighted match score for each linkage that allows for approximate field comparators and “fuzzy” matching. We experimented with different combinations of variables and different variable weighting to evaluate the linkage methods. The final matching technique used name and a ten-year age band. All matches with a probability >20% underwent clerical review by two authors (ZC and DY), including a review of case notes if necessary to assign final matches.

Two source capture-recapture analysis was conducted using the Lincoln-Petersen-Chapman estimator, to estimate the total deaths from stroke:^27^

$${\hat{N}}_{c}=\frac{\left(n+1)(K+1\right)}{k+1}-1$$

where *ˆN_c_* = Estimate of total number of fatal strokes in population; n = number of fatal strokes identified in SISLE; K = Number of fatal strokes identified in HEAL-SL; k = Number of recaptures.

Capture-recapture methods make the following assumptions^28^; however, these are frequently violated in epidemiological studies:^29^

1. That there is independence between sources. This is met in our study as the two datasets were collected independently by separate study teams.
2. That individuals captured in one dataset can be recognized as previous captures in the other dataset, that they can be clearly and uniquely identified across the two datasets. This is potentially violated in our study due to the high variation in reported ages and spelling of names in Sierra Leone, leading to undermatching of individuals across the two datasets. We therefore present an adjusted analysis accounting for undermatching.
3. That there are no false positive or false negative captures due to imperfect sensitivity and PPV of VA to identify individuals with fatal stroke. We therefore present an adjusted analysis, accounting for sensitivity of 87% and PPV of 81% of VA,^10^ to identify individuals with fatal stroke.
4. That individuals have the same probability of capture, which previous research has shown to be frequently violated in epidemiological studies.^20^ This is partially violated in our study, due to the observed selection bias of the hospital-based register. Unfortunately, due to our small sample size we are unable to stratify by covariates associated with selection bias.^30^

We present adjusted analysis due to two sources of uncertainty, high variation in reported ages and spelling of names in Sierra Leone and sensitivity and PPV of VA both of which would lead to undermatching of individuals across the two datasets. Population estimates for the Western Area were sourced from the 2021 Mid-term Population and Housing Census, with a population of 1,228,157 under the age of 70. Annual stroke deaths are presented per 100,000 population.

### Statistical analysis

Statistical analysis was conducted in STATA v17™. Selection bias on to the hospital-based register was assessed with a univariable analysis, due to the small sample size. Continuous variables, with normal distribution, were described as means and standard deviations (SD). Ordinal or non-normal variables were reported as medians and interquartile range (IQR). Pearson’s chi-square test was used to examine associations between categorical variables. Unpaired *t*-tests demonstrate associations between continuous variables and Mann–Whitney U-test was used for non-normal data.

### Ethics

Written consent was obtained for all patients in the SISLE study. Written consent was obtained from family members of all participants in the HEAL-SL dataset. Ethical approval was received from King’s College London, reference: LRS/DP-22/23-33965 and approval from the Sierra Leone Ethical and Scientific Review Committee. For HEAL-SL, ethical approval was received from the Sierra Leone Ethical and Scientific Review Committee.

## Results

**Figure 1.**
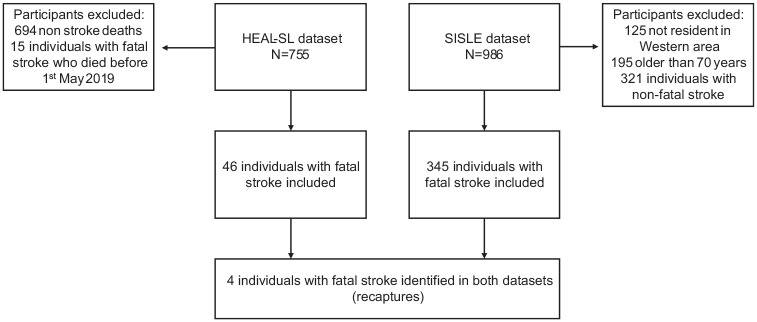
Inclusion flowchart of overlapping individuals with fatal strokes occurring in the Western Area from May 2019 to 30th September 2021 and number of individuals identified in SISLE and HEAL-SL datasets.

The HEAL-SL dataset included 755 individuals, 61 of whom had stroke as the cause of death, and 15 individuals with fatal strokes were excluded as date of death was before the inception of SISLE, leaving 46 individuals with fatal strokes maintained from the HEAL-SL dataset for analysis (Figure 1). The SISLE cohort included 986 individuals with stroke, 195 individuals were over the age of 70, a further 125 were not resident in the Western Area, and 321 did not have a fatal stroke, leaving 345 individuals with fatal strokes under the age of 70 residing within the Western Area.

**Figure 2.**
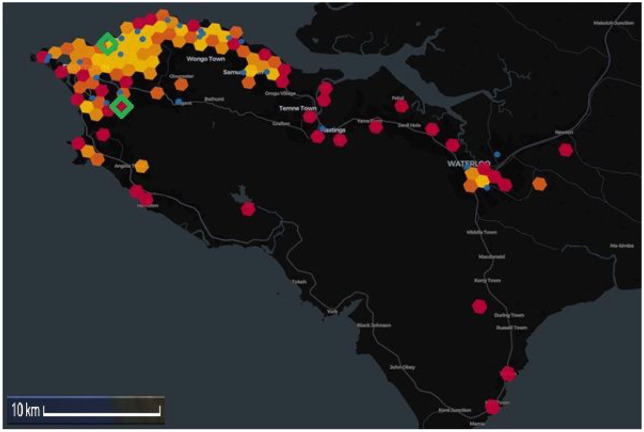
Heatmap of Western Area, Sierra Leone demonstrating participants with fatal stroke by participant address (1 km radius), hexagon heatmap indicates density of participants with fatal stroke from SISLE (red > orange > yellow) and blue dots indicate participants from HEAL-SL. Green triangles represent the two hospitals where recruitment took place.

A heatmap demonstrating the overlapping geographical locations of individuals with fatal stroke in the two datasets is shown in Figure 2.

### Selection bias of a hospital-based stroke register

Individuals in HEAL-SL had a mean age of 58 years compared to 55 years in SISLE (*p* = 0.07), 59.5% were male compared to 50.7% in SISLE (*p* = 0.28), and 52.3% had no formal education compared to 39.0% (*p* = 0.09) in SISLE, see Table 1. Individuals in HEAL-SL were more likely to be employed 36.7% vs 59.5% (*p* = 0.002), were less likely to have sought formal healthcare 48.5% vs 100% (*p* < 0.001), more likely to have died suddenly 14.3% vs 4.1% (*p* < 0.001), and less likely to have died in hospital 19.0% vs 67.5%.

**Table 1.** Univariable analysis of socio-demographics and sudden death for selection onto the SISLE hospital-based register.

Variable	SISLE only	HEAL-SL only	*p* Value
N	341	42	
**Age**	**55.1 (SD: 10.9)**	**57.8 (SD: 7)**	**0.07**
Male sex	173 (50.7%)	27 (59.5%)	0.28
No formal education (vs any)	133 (39.0%)	22 (52.3%)	0.09
**Employed (any)**	**125 (36.7%)**	**25 (59.5%)**	**0.002**
**Sought healthcare**	**341 (100%)**	**17(48.5%)** ^a^	**<0.001**
Duration of illness until death (days)	9 (4–47)	8 (2–30)	0.06
**Sudden death (yes)** ^a^ **within 24 hours**	**14 (4.1%)**	**6 (14.3%)**	**<0.001**
**Died in hospital**	**230 (67.5%)**	**8 (19.0%)**	**<0.001**

Bold text notes statistical significance at *p* < 0.05.

a This data field was only completed for 35/42 (83.3%) participants; for the remainder, it is unknown whether they sought formal healthcare or not.

For 15 out of the 17 participants who reported seeking formal healthcare in the HEAL-SL dataset and were not captured by SISLE, detailed care-seeking data were available. Ten patients visited government facilities (eight at secondary or primary health care facilities, one at Connaught Hospital and one at 34^th^ Military Hospital), four patients sought care at a private facility or from a private clinician, and one sought care from a traditional healer. While six patients in the HEAL-SL dataset alone suffered sudden deaths, the mean duration of days until death was 6.8 days (SD 7.4).

### Estimates of annual deaths from SISLE

Unadjusted and adjusted capture-recapture estimates and estimates of annual death rates from stroke are shown in Table 2. Completeness of SISLE register for fatal stroke ranged from 10.6% (95% CI: 9.6%–11.7%) to 27.2% (95% CI: 24.8%–30.0%). Annual deaths from stroke using the SISLE register alone were 11/100,000. Capture-recapture estimates of annual deaths from stroke ranged from 41 to 106/100,000.

**Table 2.** Capture-recapture estimates; unadjusted; adjusted for undermatching due to spelling/name differences; adjusted for sensitivity and PPV of VA; adjusted for both undermatching and sensitivity/PPV.

	Fatal strokes in population < 70 years	Completeness of SISLE register for fatal stroke < 70 years	Annual death rate from stroke
SISLE fatal strokes	345	-	11/100,000
Unadjusted capture-recapture	3251 (95% CI: 809–5694)	10.6% (95% CI: 9.6%–11.7%)	106/100,000
Adjusted for undermatching of 0.5	1806 fatal strokes (95% CI: 812–2800)	19.1% (95% CI: 17.3%–20.9%).	58/100,000
Adjusted for sensitivity of 87% and PPV of 81% in HEAL-SL	2283 fatal strokes (95% CI: 612–3954)	15.1% (95% CI: 13.7%–16.7%)	74/100,000
Adjusted for undermatching and sensitivity and PPV	1268 (95% CI: 606–1929)	27.2% (95% CI: 24.8–30.0%)	41/100,000

## Discussion

The SISLE hospital-based register demonstrated incomplete capture of fatal strokes in Freetown, Sierra Leone, and we demonstrate sociodemographic and clinical differences between participants with fatal stroke < 70 years between the two datasets. We estimate the hospital-based register identified between 10.6% to 27.2% of all people < 70 dying from stroke in Freetown, considerably lower than other settings. A Swedish study estimating the completeness of two hospital-based stroke registers Lund Stroke Register and Riksstroke-Lund to population estimates of incident stroke estimated the two hospital registers to be 91% and 82% complete,^12^ with similar estimates from other Northern Europe countries.^31^ This, combined with the selection bias described above, should lead to caution in extrapolating from hospital-based data to estimate population level stroke burden in our setting. Our finding are based on data from the urban capital of Sierra Leone and may not be generalisable to other regions within the country or to other LMICs with fewer health care facilities, a lower density of healthcare workers and greater barriers to care.

Estimates of annual stroke deaths in those < 70 years from the hospital-based register alone were 11/100,000. Estimates using capture-recapture methods ranged from 41 to 106/100,000, within range of the HEAL-SL VA estimates of 98.1 (89.5–107.5)/100,000. Our estimates are higher than the two modeled estimates of annual stroke deaths, the World Health Organization 2019 estimate of 48/100,000^32^ (based on vital registration data and GBD estimates) and the 2019 Global Burden of Disease (GBD) estimate of 30.09/100,00 (22.19–40.26) in < 70s^33^ (based on econometric modeling of limited stroke incidence data and vital registration data from a few African data sources).

This study assessed the selection bias and completeness of SISLE, a hospital-based stroke register in Sierra Leone, a low-income country for people under 70 years of age with fatal stroke The SISLE register attempted to address barriers to care-seeking through providing free investigations, including neuroimaging for patients with suspected stroke, and awareness raising to recruit people onto the register.^17^ However, in this study we still found evidence of selection bias onto the register. People dying of stroke identified by VA had a mean age of 58 years compared to 55 years in the hospital-based register (*p* = 0.07), similar to previous findings in Europe.^13,14^ There was a non-statistically significant difference in educational attainment, people dying of stroke identified by HEAL-SL were less likely to have received any formal education, which may reflect economic barriers to access health care or a preference for alternative care pathways such as traditional healers. Conversely, people captured only by HEAL-SL were more likely to be employed, this may reflect a difference in how this question was asked and interpreted between the two datasets, or could reflect actual health seeking differences between the two groups, with employed people potentially opting to visit private facilities over government facilities. There were more sudden deaths (within 24 hours) in HEAL-SL than in SISLE *14.3% vs 4.1% (p* *<* *0.001)* reflecting the time taken to seek care, a median of 24 hours in the SISLE dataset.^21^ 19% of participants only captured in HEAL-SL visited a primary or secondary level government health facility in the Western Area, but were not referred to the two tertiary hospitals where the SISLE register is based. This may be explained by the prohibitive cost of care at tertiary facilities and the lack of specialized stroke services during the study period. Concurrently, there may be an issue in stroke recognition by healthcare workers and communication; family members of 14 of the HEAL-SL individuals were able to report a diagnosis provided by a healthcare worker—3 were told it was a stroke, 8 were told it was hypertension, and 3 miscellaneous causes. A separate nationwide analysis of HEAL-SL reported only 19 (7%) of 275 participants who died of stroke received a diagnosis of stroke from a health professional.^34^ This could be rectified by improved stroke recognition and awareness training to the public and a targeted education campaign for nurses and junior clinicians who staff the primary and secondary care facilities. Improving stroke awareness among healthcare workers and developing referral pathways into organized stroke unit based care, now established at the Connaught Teaching Hospital, alongside reducing the cost of care and neuroimaging could support the identification and treatment of non-fatal strokes.

## Limitations

This study only focuses on people who died from stroke, it does not include people who came to hospital, received care, and survived their stroke or the many people with stroke who did not seek care at the two tertiary hospitals. Second, this current study only reports on individuals <70 years old, the incompleteness of hospital-based registers for older individuals was not quantified for Sierra Leone although evidence from Europe suggests that incompleteness of hospital-based registers may be even greater for the elderly.^13,14^

While capture-recapture methods are used frequently in epidemiology, their utility has been questioned.^29^ Capture-recapture models rely on three assumptions.^28^ First, that the two sources are independent, which was met in our study as our two studies were conducted independently by separate study teams. Second, that individuals captured in one dataset can be recognized as previous captures in the other dataset. This is potentially violated in our study due to the high variation in reported ages and spelling of names in Sierra Leone, leading to undermatching of individuals across the two datasets. We therefore present an adjusted analysis accounting for undermatching at an arbitrary rate of 0.5. It is potentially further violated due to imperfect sensitivity and PPV of VA to identify individuals with fatal stroke. We therefore present an adjusted analysis, accounting for sensitivity of 87% and PPV of 81% of VA to identify individuals with fatal stroke. Third, that all individuals have the same probability of capture. This assumption is frequently violated in epidemiological studies^30^ and was violated in our study, due to the observed selection bias of the hospital-based register. We were only able to conduct two source capture-recapture, which prevented inclusion of covariates or modeling,^30^ which are potential methods to account for the violation of this assumption. The small number of recaptures (n = 4), led to wide uncertainty in our estimates, we attempted to mitigate this through use of the Lincoln-Petersen-Chapman estimator which is more robust to small sample sizes. The wide range of estimates provided in Table 2 demonstrate the inherent uncertainty in our estimates, due to undermatching, imperfect sensitivity of VA methods, and our small sample size of recaptures.

### Strengths

There is an absence of primary data sources on stroke burden in Sierra Leone, and this publication attempts to provide estimates of annual stroke deaths for decision-makers. We highlight the variation between estimates of deaths from stroke reported by hospital-based registers, hospital-based registers adjusted for completeness, to VA estimates and GBD models. Researchers and policy makers should carefully consider the data source and estimates chosen. Our study underlines the significant barriers in access to care for stroke, highlights some groups who are less likely to access care, and suggests the need for stroke awareness and recognition training for the health workforce.

## Conclusions

Hospital-based stroke registers underestimate total deaths from stroke for people under 70 years of age in our setting to a greater degree than previously reported in high-income countries. For people under 70 years of age dying from SISLE, employed people, people who did not seek formal healthcare, and people who died within 24 hours were less likely to be included in the hospital-based stroke register. Investment in routine death registration systems and population-based stroke surveillance is essential to provide essential data on the population burden of SISLE.
